# CHNQD-00603 Promotes Osteogenic Differentiation of Bone Marrow Mesenchymal Stem Cells by the miR-452-3p-Mediated Autophagy Pathway

**DOI:** 10.3389/fcell.2021.779287

**Published:** 2021-12-21

**Authors:** Shanshan Xin, Shao-Ming Li, Ling Gao, Jing-Jing Zheng, Yan-Wei Wu, Chang-Lun Shao, Wen-Hao Ren, Keqian Zhi

**Affiliations:** ^1^ Department of Oral and Maxillofacial Surgery, The Affiliated Hospital of Qingdao University, Qingdao, China; ^2^ School of Stomatology, Qingdao University, Qingdao, China; ^3^ Key Lab of Oral Clinical Medicine, The Affiliated Hospital of Qingdao University, Qingdao, China; ^4^ Department of Endodontics, The Affiliated Hospital of Qingdao University, Qingdao, China; ^5^ Key Laboratory of Marine Drugs, The Ministry of Education of China, School of Medicine and Pharmacy, Ocean University of China, Qingdao, China

**Keywords:** osteogenesis, marine natural products, periodontitis, miRNAs, autophagy

## Abstract

**Background:** Periodontitis is a chronic and progressive disease accompanied by bone loss. It is still a challenge to restore the bone structure. The osteogenic differentiation of bone marrow mesenchymal stem cells (BMSCs) plays a decisive role in bone restoration and regeneration. Marine natural products (MNPs) have multiple biological activities, including anti-tumor and anti-inflammatory properties. However, the exploration of MNPs in osteogenesis is far from sufficient.

**Methods:** We obtained a series of derivatives through structural optimization from 4-phenyl-3,4-dihydroquinolin-2(1H)-one alkaloid isolated from *Scopulariopsis* sp. Some preliminary cytological experiments showed that CHNQD-00603, obtained by adding a methoxy group to the position C3 and a hydroxyl group to the position C4 of 4-phenyl-3,4-dihydroquinolin-2(1H)-one, might promote the osteogenic differentiation of BMSCs. To further investigate the effects of CHNQD-00603 on BMSCs, we performed a CCK-8 assay and qRT-PCR, alkaline phosphatase staining (ALP), and alizarin red S staining to assess the cytotoxicity and the ability of osteogenic differentiation of CHNQD-00603. The autophagy level was assessed and validated by WB, qRT-PCR, and transmission electron microscopy. Then, 3-methyladenine (3-MA) was added to further examine the role of autophagy. Based on the expression of autophagy-related genes, we predicted and examined the potential miRNAs by bioinformatics.

**Results:** CCK-8 assay showed that CHNQD-00603 at 1 µg/ml did not influence BMSCs activity. However, the proliferation rate decreased from the seventh day. qRT-PCR, ALP staining, ALP activity assay, and Alizarin red S staining showed that the best concentration of CHNQD-00603 to promote osteogenic differentiation was 1 µg/ml. Further investigations indicated that CHNQD-00603 activated autophagy, and the inhibition of autophagy by 3-MA attenuated CHNQD-00603-enhanced osteogenic differentiation. Subsequently, the findings from bioinformatics and qRT-PCR indicated that miR-452-3p might be a regulator of autophagy and osteogenesis. Furthermore, we transfected BMSCs with miR-452-3p NC and mimics separately to further determine the function of miR-452-3p. The data showed that the overexpression of miR-452-3p moderated the level of autophagy and osteogenic differentiation of CHNQD-00603-treated BMSCs.

**Conclusion:** Our data suggested that CHNQD-00603 promoted the osteogenic differentiation of BMSCs by enhancing autophagy. Meanwhile, miR-452-3p played a regulatory role in this process.

## Introduction

Periodontitis and peri-implantitis are frequent oral diseases and a significant cause of tooth loss and implant failure (Smeets et al., 2014; Cardoso et al., 2018). In addition, they severely affect the quality of patients’ lives. At present, the treatment of periodontitis and peri-implantitis mainly focuses on prevention, basic treatment, and guided tissue regeneration (Jepsen et al., 2015; Graziani et al., 2017). BMSCs with low immune response are a key element of bone regeneration that can differentiate into osteoblasts, chondrocytes, and adipocytes in different microenvironments (Kokabu et al., 2016). Transplantation of stem cells by injecting cell suspensions or seeding cells onto scaffolds has been demonstrated to promote bone regeneration *in vivo* in animals (Sinder et al., 2020; Jiang et al., 2021). More importantly, studies have investigated BMSCs in periodontal tissue regeneration (Ledesma-Martínez et al., 2019). However, the challenge is that the limited ability of osteogenic differentiation and rapid aging limits their clinical application (de Witte et al., 2017). Recently, scholars have focused on the discovery of bioactive compounds that can induce osteogenic differentiation of BMSCs (Ringe et al., 2002). Marine natural products (MNPs) are an excellent resource due to their diversity and abundance.

**FIGURE 1:**
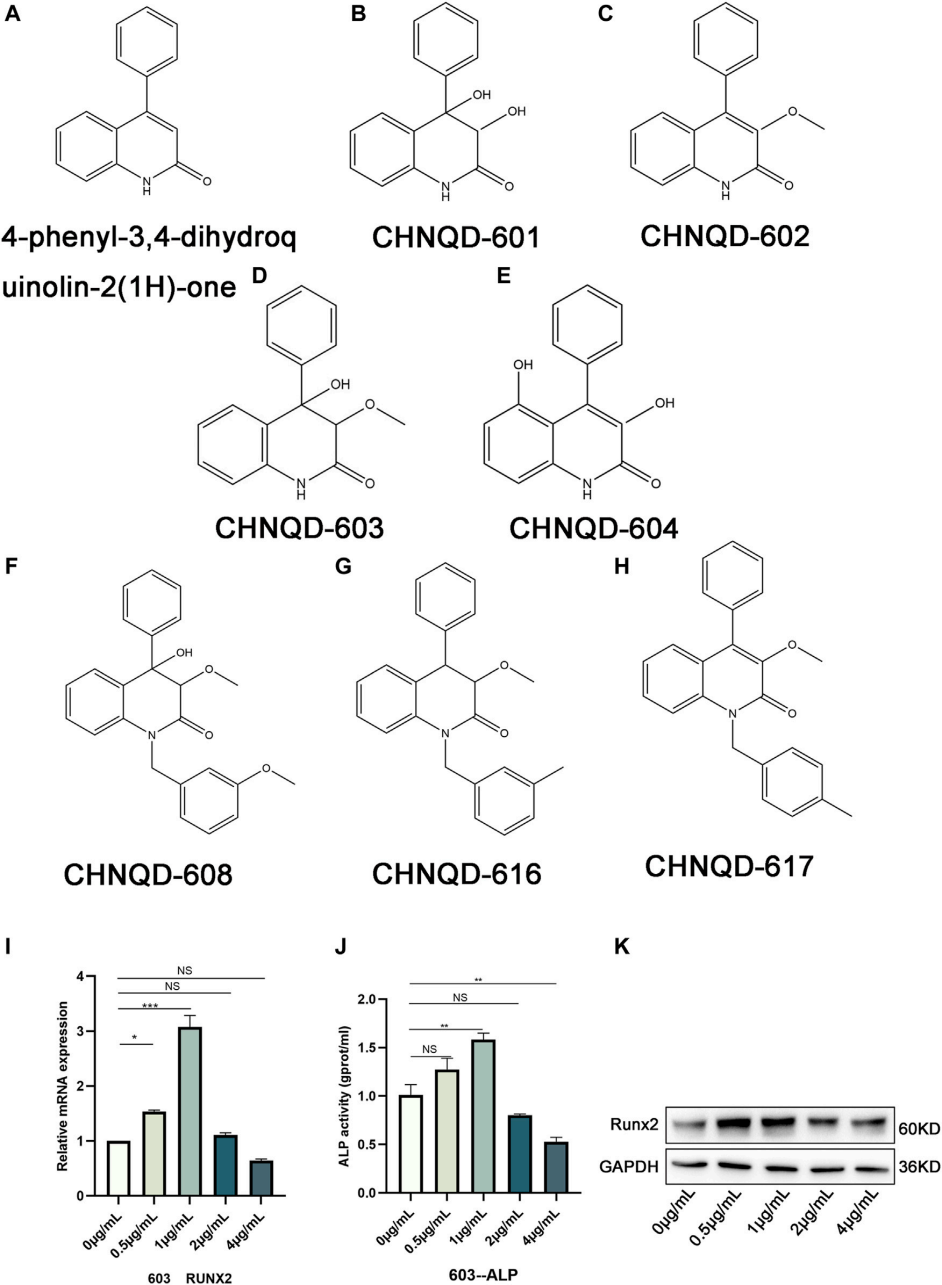
The structure of 4-phenyl-3,4-dihydroquinolin-2(1H)-one and its derivatives. **(A)** The structure of 4-phenyl-3,4-dihydroquinolin-2(1H)-one. **(B–H)** Structures of a class of 4-phenyl-3,4-dihydroquinolin-2(1H)-one alkaloid derivatives. **(I)** qRT-PCR was used to observe the expression of the osteogenesis-related genes Runx2. **(J)** ALP activity assay was applied to access the early osteogenic differentiation of BMSCs. **(K)** WB was adopted to investigate the osteogenesis-related protein Runx2. Quantitative data were presented as the mean ± SD (n = 3) (*p < 0.05, **p < 0.01, ***p < 0.0001).

Marine natural products (MNPs) from marine organisms provide abundant and promising resources for bone research and, have been reported to be closely associated with bone growth and healing (Carson and Clarke, 2018). In the early stage, our cooperative team isolated a series of 4-phenyl-3,4-dihydroquinolin-2(1H)-one alkaloids from a gorgonian-derived fungus, i.e., *Scopulariopsis* sp. (TA01-33) (Shao et al., 2015). In this study, we obtained various new derivatives by adding different functional groups to 4-phenyl-3,4-dihydroquinolin-2(1H)-one core. The structure of some derivatives is presented in Figures 1A–H. Some preliminary cytological experimental results from osteogenesis-related gene expression, alkaline phosphatase activity (ALP), and western blotting both showed that CHNQD-00603, one of the derivatives, could promote osteogenic differentiation of BMSCs (Figures 1I–K). Therefore, CHNQD-00603 might be an effective molecule in osteogenesis. However, its mechanism of action is still unclear. Therefore, the present study aimed to further determine the effect of CHNQD-00603 on osteogenic differentiation and the relevant mechanisms.

Autophagy plays an essential role in maintaining the energy and nutrient balance by recycling unnecessary or damaged organelles and proteins (Wong et al., 2020). In the past few years, significant progress has been made in understanding the mechanisms by which autophagy participates in life activities (Glick et al., 2010; Parzych and Klionsky, 2014). mTOR signaling pathway is believed to be associated with autophagy (Al-Bari and Xu, 2020). Interestingly, in the present study, the mTOR signaling pathway and PI3K-AKT signaling, which participate in autophagy regulation, were predicted to be associated with CHNQD-00603 (Figure 3F).

In this study, we evaluated the cytotoxicity of CHNQD-00603 and its ability to promote osteogenic differentiation of BMSCs. First, we predicted the possible target genes of CHNQD-00603 by SwissTargetPrediction according to the structure. The results are presented in Table 1. DAVID analysis indicated that the predicted gene of this derivative was connected to the signaling pathways of bone metabolism and autophagy, including signaling pathways regulating pluripotency of stem cells, osteoclast differentiation, VEGF signaling pathways, and mTOR signaling pathway (Figure 3F). Our data confirmed that CHNQD-00603 promoted the osteogenic differentiation of BMSCs. Next, we further found that the effect was regulated by the miR-452-3p-mediated autophagy pathway.

**TABLE 1:** Probable predictive target genes.

—	—	—	—
CYP3A4	EWS-Fli1	EIF2AK3	MAPK10
CYP2C19	ADRA1D	KDM5C	AHCY
CA2	EDNRA	KDM4B	ALOX15
CA1	PSMB5	KDM5B	SLC5A1
TOP2A	BDKRB1	KDM4A	PFKFB4 PFKFB3
CES2	JAK3	CSNK2A1	HMGCR
JAK1	TYK2	CA7	FLT1
JAK2	PDE10A	CA6	KIT
CASP1	ABCC9	CA12	KDR
NFKBIA	LRRK2	CA14	MMP1
RELA	EDNRB	CA9	MMP2
ERBB2	FBP1	CA4	MMP8
EGFR	ADRA2C	CA13	DHODH
AOC3	CDK5R1 CDK5	CA5B	ADORA1
CSF1R	ALOX12	CA5A	PTAFR
ACPP	CYP51A1	CDK2 CCNA1 CCNA2	CDC7
HSD17B2	CAPN1	CDK9 CCNT1	—

## Materials and Methods

### Isolation and Culture of Bone Marrow Mesenchymal Stem Cells (BMSCs)

Two-month-old female Sprague-Dawley rats were used to isolate the BMSCs. The rats were anesthetized with 10% chloral hydrate and sacrificed. The femur and tibia were collected after disinfection with alcohol. Bone marrows from the femur and tibia of the rats were flushed out by α-minimum essential medium (α-MEM) under aseptic conditions. After centrifuging, BMSCs were transferred to culture flasks. The medium was changed every 3 days until the adherent cells grew to 80% of the culture flask. BMSCs at passage three were used to estimate the surface markers, including CD45, CD90, CD29, and CD11b/c (Elabscience, China) by flow cytometry. All the experimental protocols were approved by the Intramural Animal Use and Care Committee of the affiliated hospital of Qingdao University.

### Cell Counting Kit-8 (CCK-8) Analysis

The third-generation cells were seeded into a 96-well plate (7,000 cells/well) and cultured for 24 h. Then the medium was replaced with 100 µL of new medium added with different concentrations of CHNQD-00603 (0 µg/ml, 0.5 µg/ml, 1 µg/ml, 2 µg/ml, and 4 µg/ml). The medium was changed every 3 days. After culturing for 3, 5, and 7 days, the cell proliferation and cell viability of BMSCs were examined by a CCK-8 detection kit following the manufacturer’s instructions.

### Osteogenic Differentiation of BMSCs

The third-generation cells were placed in a six-well plate (2×10^5^ cells/well). The medium was replaced with osteogenic induction medium, consisting of α-MEM supplemented with 10% fetal bovine serum (HyClone, United States), 1% penicillin and streptomycin (Solarbio, China), dexamethasone (10 nM, Solarbio), beta-Glycerol phosphate (10 mM, Solarbio) and ascorbic acid 2-phosphate (200 μM, Solarbio) when the cell density reached 80% of the plate. The medium was changed every 3 days. ALP staining (Meilunbio, China) was performed 7 days later to observe the early osteogenic differentiation. Moreover, after 14 days, ARS (Solarbio, China) was used to assess late osteogenic differentiation according to the manufacturer’s instructions. The staining was observed under a microscope at low magnification.

### Adipogenic and Chondrogenic Differentiation of BMSCs

The third-generation cells were placed in a six-well plate (2×10^5^ cells/well). The adipogenic differentiation medium (Procell, China) and chondrogenic differentiation medium (Procell, China) were added, respectively, according to the manufacturer’s instructions after the cell density reached 80% of the plate. The medium was changed every 3 days. After 14 days, Oil Red O staining assay (VivaCell Bioscience) and Chondro-dye (VisaCell Biosciences) were used to observe the adipogenic differentiation and adipogenic differentiation under a microscope at low and high magnification.

### RNA Extraction and Quantitative Real-Time PCR (qRT-PCR) Analysis

Total RNA was obtained from the BMSCs using Trizol reagents. A PrimeScript RT (TaKaRa) reagent kit was applied to synthesize the cDNA. qRT-PCR was conducted by a CFX96 Real-Time System (BIO-RAD) using SYBR Premix Ex Taq (TaKaRa). The expression of mRNA was normalized to GAPDH or U6 using the 2^−ΔΔCt^ method. The primers are listed in Table 2.

**TABLE 2:** The sequence of primers.

Gene	Sequence
Runx2	Forward:CTTCAAGGTTGTAGCCCTCG
	Reverse:TAGTTCT CATCATTCCCGGC
ALP	Forward:CTAGTTCCTGGGAGATGGTA
	Reverse:GTGTTGTACGTCTTGGAGAGA
OCN	Forward:CATGAGGACCCTCTCTCTGC
	Reverse:TGGACATGAAGGCTTTGTCA
ATG5	Forward:TGGGATTGCAAAATGACAGA
	Reverse:TTCCCCATCTTCAGGATCAA
LC3	Forward:TACCAAGGCAAAAAGGGACG
	Reverse:CCCCTGACACTGCTCTTCTAT
P62	Forward:AGCTGCCCTCAGCCCTCT
	Reverse:GGCTTCTCTTCCCTCC
GAPDH	Forward:CCTCGTCTCATAGACAAGATGGT
	Reverse:GGGTAGAGTCATACTGGAACATG
ATG14	Forward:TGCCGAACAATGGGGACTAC
	Reverse:AGGCAG GGTTGTTATGCTCC
Atg7	Forward:TTTGTGGACAACGCCAAGATC
	Reverse:GAACCCGCTGGCATTCACT
Atg12	Forward:ACCCGGACTGTCCAAGCA
	Reverse:ACCATCACTGCCAAAACACTCA
Atg16L1	Forward:CATGGACCGCAGGGTTAAAC
	Reverse:CGGCTTGCAAAATCATTTGA
Beclin1	Forward:CCAGACAGTGTTGTTGCTCCAT
	Reverse:CGCAAACCCCCAGAACAGTA
rno-miR-452-3P	Forward:GGCCTCAGTCTCATCTGCAAA
	RT:GTCGTATCCA​GTG​CAG​GGT​CCG​AGG​TATTCGCACTGGATACGACCTTCTT
rno-miR-6331	Forward:GGGCTTTGGTGGCTTAGTTCTTT
	RT:GTCGTATCCAGTGCAGGGTCCGAGGTATTCGCACTGGATACGACGCACAA
U6	Foward:CGCTTCGGCAGCACATATACTA
	RT:GGAACGCTTCACGAATTTGC
Universe R	CCAGTGCAGGGTCCGAGGT

### Western Blot

The specific methods have been introduced in detail in previous articles (Zhang et al., 2021). In brief, the cells were lysed with cell lysis buffer for Western and IP (Beyotime Biotechnology) supplemented with PMSF and cocktail on ice for 30 min. After centrifugation, the cell lysates were collected and analyzed with BCA Protein Assay Kit (Solarbio). Equal amounts of protein were added to SDS-containing polyacrylamide gels and then transferred to polyvinylidene difluoride (PVDF) membranes (Sigma-Aldrich, China). 5% skimmed milk was utilized to block the membrane at room temperature for 2 h. Then the primary monoclonal antibodies were incubated with the membranes overnight at 4°C. Excessive antibodies were removed with TBST. Then, species-specific secondary antibodies were used to bind to the corresponding primary antibodies for 1 h at room temperature. The ChemiDoc Touch Imaging System (BioRad) was applied to collect images, and the ImageJ was used to quantify proteins.

### Alizarin Red S Staining, ALP Activity Assay, and ALP Staining

Alizarin Red solution (Solarbio, China) was applied to detect the effect of CHNQD-00603 on the osteogenic differentiation ability of BMSCs. The third-generation BMSCs were placed in a six-well plate (2×10^5^ cells/well). The different concentrations of CHNQD-00603 solutions were respectively added to cells after the cell density reached 80%. The medium was changed every 3 days. In addition, 3-MA (an inhibitor of autophagy), Rapa (an inhibitor of the mTOR pathway), and miR-452-3p were added in different experiments. After osteogenesis induction for 14 days, the cells were fixed by 4% paraformaldehyde and stained with Alizarin Red solution following the manufacturer’s instructions. In addition, ALP activity assay and ALP staining were used to detect the early osteogenic differentiation. After culturing in the osteogenesis-inducing media for 7 days, the cells were detected and stained by ALP activity (Beyotime, China) and an alkaline phosphatase kit (Meilunbio, China).

### Prediction of Probable Target Genes and Signaling Pathway Analysis

Swiss TargetPrediction database (http://www.swisstargetprediction.ch/) was used to predict the possible target genes according to CHNQD-00603 structure. DAVID database (https://david.ncifcrf.gov/summary.jsp) was used to evaluate the probable signaling pathway by the predicted target genes.

### Prediction of miRNA

miRDB database ((http://mirdb.org/) was applied to find the potential miRNA that could bind to the changed autophagy-related genes (Atg5 and Atg14). Cytoscape 3.6.1 was applied to draw the final visualization.

### Transmission Electron Microscopy

BMSCs (2×10^5^ cells/well) were treated with or without 1 µg/ml CHNQd-00603 for 7 days, or BMSCs (2×10^5^ cells/well) were transfected with miR-452-3p NC and mimics for 7 days. Then, the treated cells were fixed in 2.5% glutaraldehyde (Solarbio). The next steps were performed by Servicebio Company, and a transmission electron microscope was used to observe the results.

### Immunofluorescence Analysis

The pretreated cells were spread into small confocal dishes. After 48 h, the cells were fixed by 4% paraformaldehyde and permeabilized with 0.1% Triton X-100 for 10 min. Then, 5% bovine serum albumin (FBS) was used to block the cells, and then LC3 (1:200, Proteintech) antibody was used to incubate the cells at 4°C overnight. The next day, the cells were incubated with the second antibody for 1 h, and the nucleus was stained with DAPI (Sigma-Aldrich) for 5 min. A laser scanning confocal microscope was used to observe the images.

### Cell Transfection

Third-passage BMSCs were transfected with negative control (NC) and miR-452-3p (mimics) by Lipofectamine 3,000 (Invitrogen, United States) according to the manufacturer’s instructions. After transfecting for 48 h, transfection efficiency was determined by qRT-PCR.

### Statistical Analysis

All the corresponding experiments were independently repeated three times. The data were expressed in mean ± SD and analyzed by *t*-test and one-way ANOVA with Graphpad Prism 8.0. Bonferroni test was used as a post hoc test when the results analyzed by one-way ANOVA were significant. *p* < 0.05 was considered statistically significant.

## Results

### The structures of 4-phenyl-3,4-dihydroquinolin-2(1H)-one alkaloid derivatives

Figure 1 represents the structures of 4-phenyl-3,4-dihydroquinolin-2(1H)-one alkaloid and some of its derivatives. Figure 1A presents the structure of 4-phenyl-3,4-dihydroquinolin-2(1H)-one core. CHNQD-00601 was obtained by adding a hydroxyl group to the position C3 and position C4 of 4-phenyl-3,4-dihydroquinolin-2(1H)-one core (Figure 1B). CHNQD-00602 was obtained by adding a methoxy group to the position C3 of 4-phenyl-3,4-dihydroquinolin-2(1H)-one core (Figure 1C). CHNQD-00603 was gained by adding a methoxy group to the position C3 and a hydroxyl group to the position C4 of 4-phenyl-3,4-dihydroquinolin-2(1H)-one (Figure 1D). CHNQD-00604 was obtained by substituting hydrogen at positions C3 and C5 with hydroxyl groups (Figure 1E). The derivative CHNQD-00608 was formed by replacing the hydrogen of position N1, position C3, and position C4 with 1-(3-methoxy benzyl), methoxy, and hydroxyl groups, respectively (Figure 1F). The derivatives CHNQD-00616 and CHNQD-00617 both replaced hydrogen with a methoxy group at position C3, but the position N1 of CHNQD-00616 was 1-(3-methyl benzyl) while CHNQD-00617 was 1-(4-methyl benzyl) (Figures 1G, H). The results of some preliminary cytological experiments indicated that the expression of osteogenesis-related gene Runx2, ALP activity, and osteogenesis-related protein Runx2 had the same trend (Figures 1I–K). Therefore, we believe that CHNQD-00603 could promote osteogenic differentiation, and we chose CHNQD-00603 for further investigation.

### The Effects of CHNQD-00603 on Cell Viability and Osteogenic Differentiation of BMSCs

**FIGURE 2:**
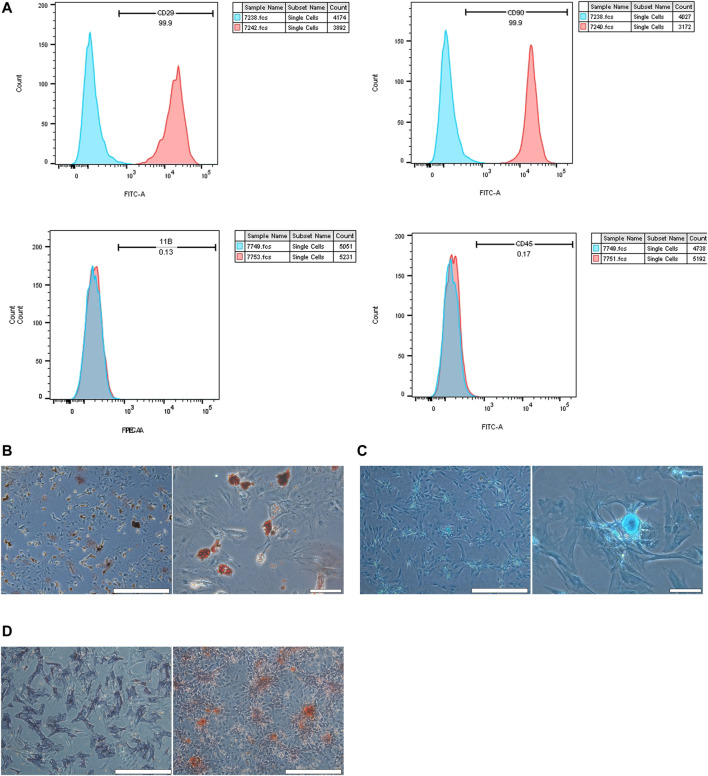
Validation of BMSCs. **(A)** flow cytometry was used to confirm the characterization of BMSCs. BMSCs expressed CD90 and CD29, but they did not express CD45 and CD11b/c. **(B)** Adipogenic differentiation was identified by Oil Red O staining at low and high magnification. **(C)** Chondrogenic differentiation was assessed by chondroblast staining at low and high magnification. **(D)** Osteogenic differentiation was evaluated by ALP staining and ARS staining at low magnification (scale bar = 200 µm).

**FIGURE 3:**
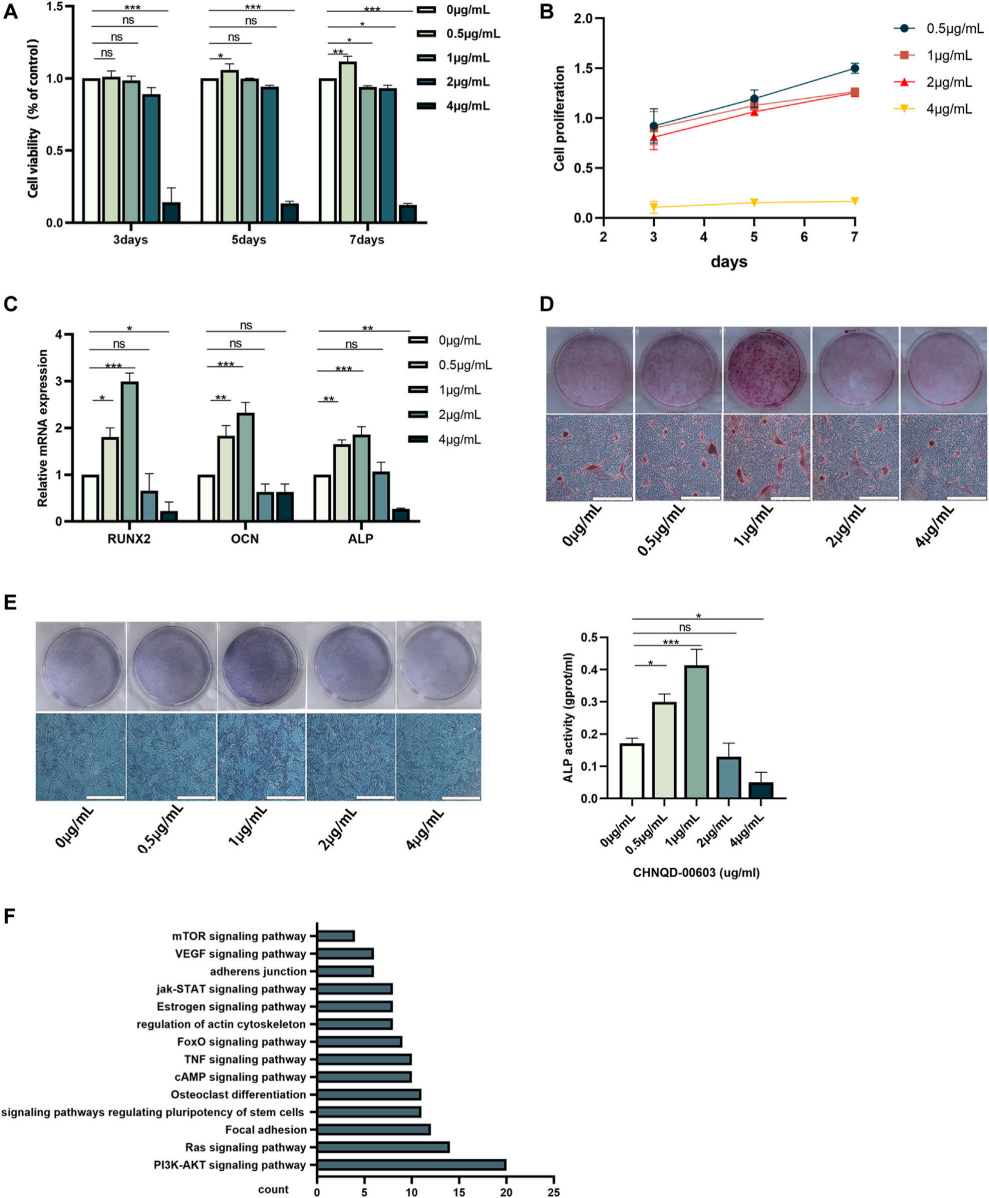
The effect of different concentrations of CHNQD-00603 on cell viability and osteogenic differentiation of BMSCs. **(A, B)** CCK-8 assay was performed to detect cell viability and cell proliferation of BMSCs cultured with different concentrations of CHNQD-00603 (0 µg/ml, 0.5 µg/ml, 1 µg/ml, 2 µg/ml, and 4 µg/ml) for 3, 5, and 7 days **(C)** qRT-PCR tested the expression of osteogenesis-related genes in CHNQD-00603-induced BMSCs on day 7. **(D)** Alizarin red S staining indicated calcium salt deposits on day 14. **(E)** ALP staining and ALP activity were evaluated on day 7 (scale bar = 200 μm, scar bar = 200 µm). **(F)** Bioinformatic analysis of predicted target genes. Quantitative data are presented as the mean ± SD (n = 3) (*p < 0.05, **p < 0.01, ***p < 0.0001).

BMSCs isolated from the bone marrow were positive for CD29 and CD90 and negative for CD45 and CD11b/c (Figure 2A). The adipogenic differentiation and chondrogenic differentiation were observed at low and high magnification, respectively. The results showed that fat cells were stained orange, and chondrocytes were stained blue (Figures 2B, C). ALP staining and ARS at low magnification indicated the formation of osteoblasts (Figure 2D). To estimate the response of BMSCs to CHNQD-00603, we treated the cells with different concentrations of CHNQD-00603 (0 µg/ml, 0.5 µg/ml, 1 µg/ml, 2 µg/ml, and 4 µg/ml) and then detected the cell viability and cell proliferation with a CCK-8 reagent. After incubating for 5 and 7 days, CHNQD-00603 was shown to promote cell proliferation at a dose of 0.5 µg/ml compared with the untreated group. CHNQD-00603 did not affect cell proliferation at 1 µg/ml and 2 µg/ml after 3 and 5 days, but the cell proliferation rate began to decrease on day 7. However, CHNQD-00603 exhibited a noticeable inhibitory effect at a concentration of 4 µg/ml (Figures 3A, B). Subsequently, we examined the osteogenic differentiation of BMSCs treated with different doses of CHNQD-00603 after 7 days. Osteogenesis-related gene was determined by qRT-PCR. The results showed that the mRNA expression of alkaline phosphatase (ALP), osteocalcin (OCN), and Runt-related transcription factor 2 (RUNX2) increased at concentrations 0.5 µg/ml and 1 µg/ml, with 1 µg/ml showing the strongest effect on mRNA expression, which was two to three folds compared with the untreated group. Besides, there was no effect or inhibitory effect on mRNA expression at 2 and 4 µg/ml, respectively (Figure 3C). To further determine the effect of CHNQD-00603 on osteogenic differentiation, Alizarin red S staining (ARS) was analyzed. The results showed more calcium salt deposits at a concentration of 1 µg/ml than other concentrations on day 14 (Figure 3D). In addition, ALP activity, which indicated the early osteogenic differentiation of BMSCs, was assessed by staining and quantitative analysis after 7 days. Consistent with mRNA expression and ARS staining, there was a more significant ALP staining and a higher ALP quantitation at the concentration of 1 µg/ml (Figure 3E) and a lower increase at 0.5 µg/ml. Taken together, CHNQD-00603 exhibited no cytotoxicity to BMSCs but promoted osteogenic differentiation of BMSCs at a dose of 1 µg/ml. Therefore, we selected 1 µg/mL as a proper concentration for further experiments.

### CHNQD-00603 Activates Autophagy in the Osteogenic Differentiation of BMSCs

**FIGURE 4:**
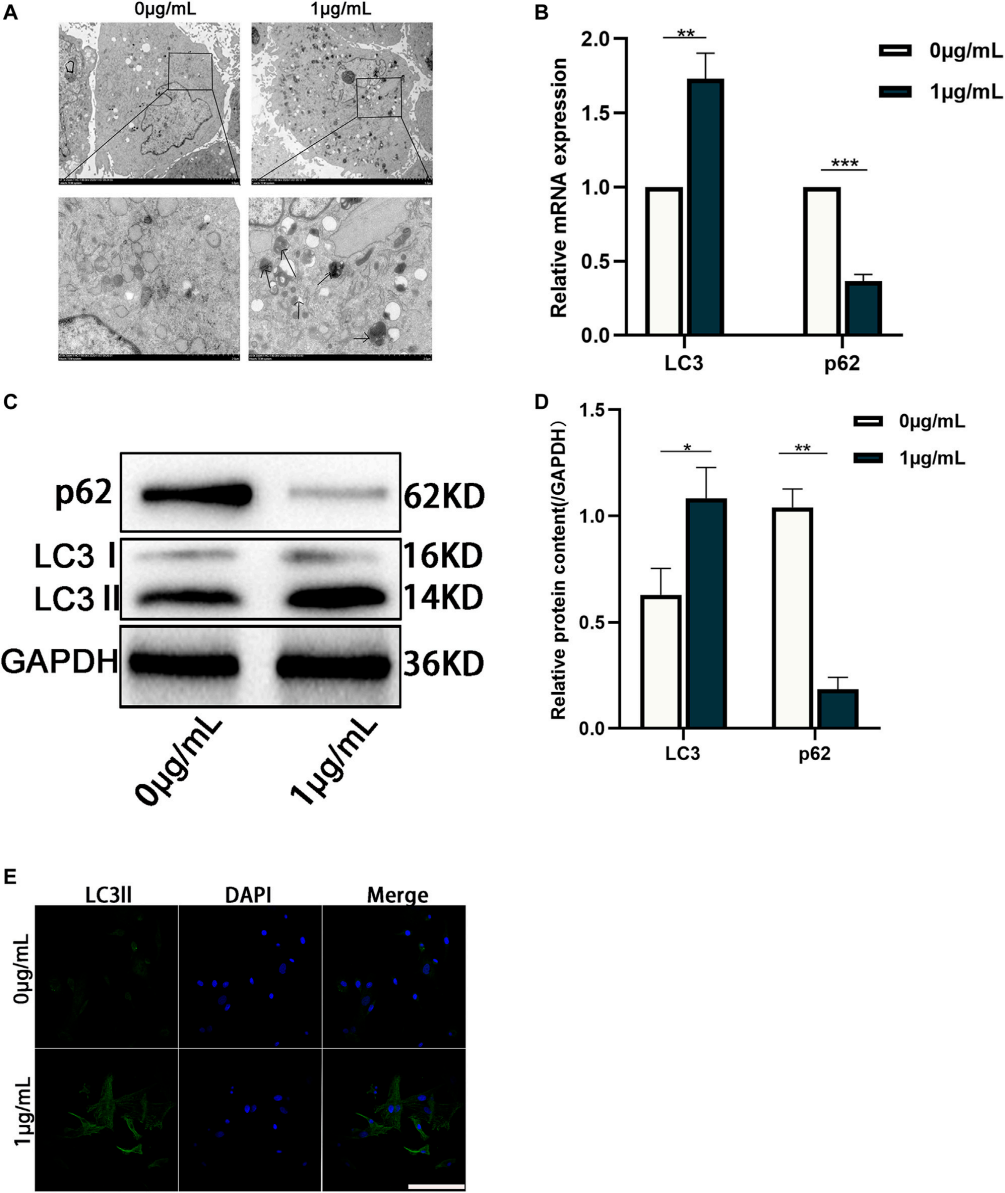
Autophagy is activated in the osteogenic differentiation of BMSCs treated with CHNQD-00603. **(A)** TEM was used to observe the number of autophagosomes on day 7. **(B)** qRT-PCR was performed to detect the expression of autophagy-related gene LC3 and p62. **(C,D)** Western blot and quantitation were performed to analyze the expression of proteins LC3 and p62. **(E)** Immunofluorescence staining was used to observe the distribution of LC3 (scale bar = 50 µm). Quantitative data are presented as the mean ± SD (n = 3) (**p < 0.01, ***p < 0.0001).

According to our prediction and analyses, autophagy-related signaling pathways-mTOR signaling pathway and PI3K-AKT signaling pathway might be associated with CHNQD-00603, which indicated that autophagy might be involved in the osteogenic differentiation of BMSCs treated with CHNQD-00603 (Figure 3F). Thus, we investigated the autophagy level in BMSCs treated with CHNQD-00603. Transmission electron microscopy (TEM) was used to observe autophagosomes. Compared with the untreated group, CHNQD-00603 significantly increased the number of autophagosomes in BMSCs on day 7 of osteogenesis (Figure 4A). QRT-PCR was then applied to detect the expression of autophagy-related genes (LC3 and p62). The results showed that the autophagy-related gene LC3 increased about 1.7-fold that in the untreated group. In addition, the expression of p62 was 0.3-fold that in the untreated group (Figure 4B). The autophagy-related protein LC3 was tested by western blot and quantitative assay and immunofluorescent assays to further estimate whether autophagy was elevated. We found that the expression of the autophagy-related protein LC3 increased in BMSCs treated with CHNQD-00603, and p62 was attenuated (Figures 4C, D). Accordingly, more LC3 dots were accumulated in BMSCs treated with CHNQD-00603 compared with the untreated group (Figure 4E). These data suggested that CHNQD-00603 activated autophagy.

### CHNQD-00603 Promotes Osteogenic Differentiation of BMSCs by Upregulating Autophagy

**FIGURE 5:**
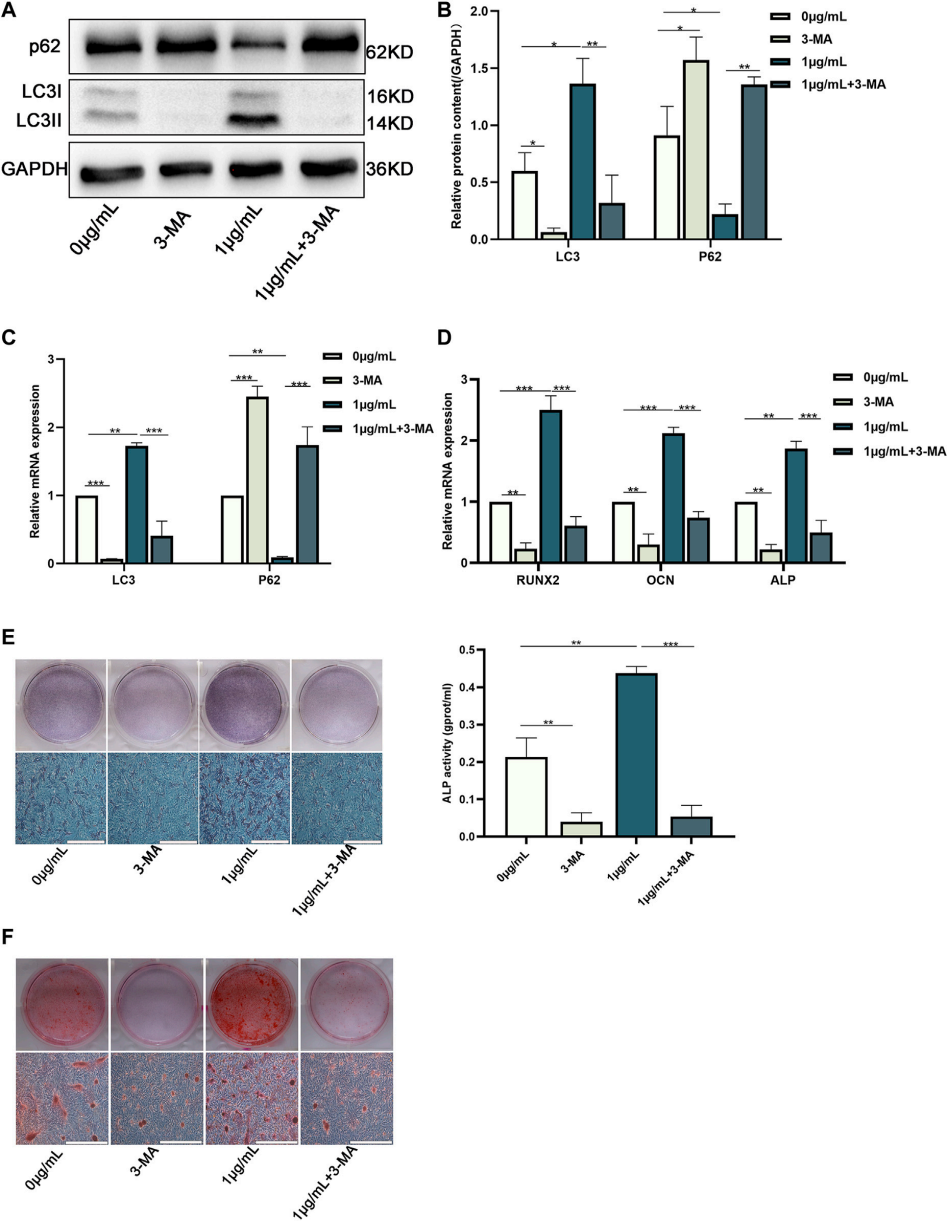
Downregulation of autophagy inhibited the osteogenic differentiation of BMSCs treated with CHNQD-00603. BMSCs cultured with or without CHNQD-00603 were treated with 5-µM 3-MA and then induced for osteogenic differentiation. The level of autophagy and osteogenesis was analyzed on day 7. **(A,B)** Autophagy-related proteins LC3 and p62 were detected by Western blot and quantitation. **(C)** Autophagy-related genes LC3 and p62 were assessed by qRT-PCR. **(D)** Osteogenesis-related genes Runx2, OCN, and ALP were tested by qRT-PCR. **(E)** ALP staining and ALP activity assay indicated early osteogenic differentiation (scale bar = 200 µm). **(F)** ARS was used to detect calcium salt deposits on day 14 (scale bar = 200 µm). Quantitative data are presented as the mean ± SD (n = 3) (*p < 0.05, **p < 0.01, ***p < 0.0001).

3-methyladenine (3-MA, an inhibitor of autophagy) was added to the control and CHNQD-00603 groups to change autophagic activity to examine the role of autophagy in CHNQD-00603-induced osteogenic differentiation of BMSCs. The results of western blotting and quantitative assay and qRT-PCR suggested that 5-μM 3-MA decreased the level of autophagy-related protein and mRNA of LC3 in the two groups, while the expression of p62 was enhanced (Figures 5A–C). Next, we further tested whether CHNQD-00603 promoted osteogenic differentiation by autophagy. QRT-PCR data showed that the expression of osteogenesis-related mRNAs (RUNX2, OCN, and ALP) was consistent with the level of autophagy. In particular, 3-MA attenuated CHNQD-00603-induced expression of osteogenesis-related genes (Figure 5D). Consequently, ALP staining and ALP activity indicated that the early osteogenic differentiation induced by CHNQD-00603 was inhibited by adding 3-MA (Figure 5E). ARS staining demonstrated that 3-MA decreased calcium salt deposits (Figure 5F). These findings revealed that autophagy plays an indispensable role in the CHNQD-00603-induced osteogenic differentiation.

Prediction and validation of miRNAs in CHNQD-00603-induced osteogenic differentiation of BMSCs.

**FIGURE 6:**
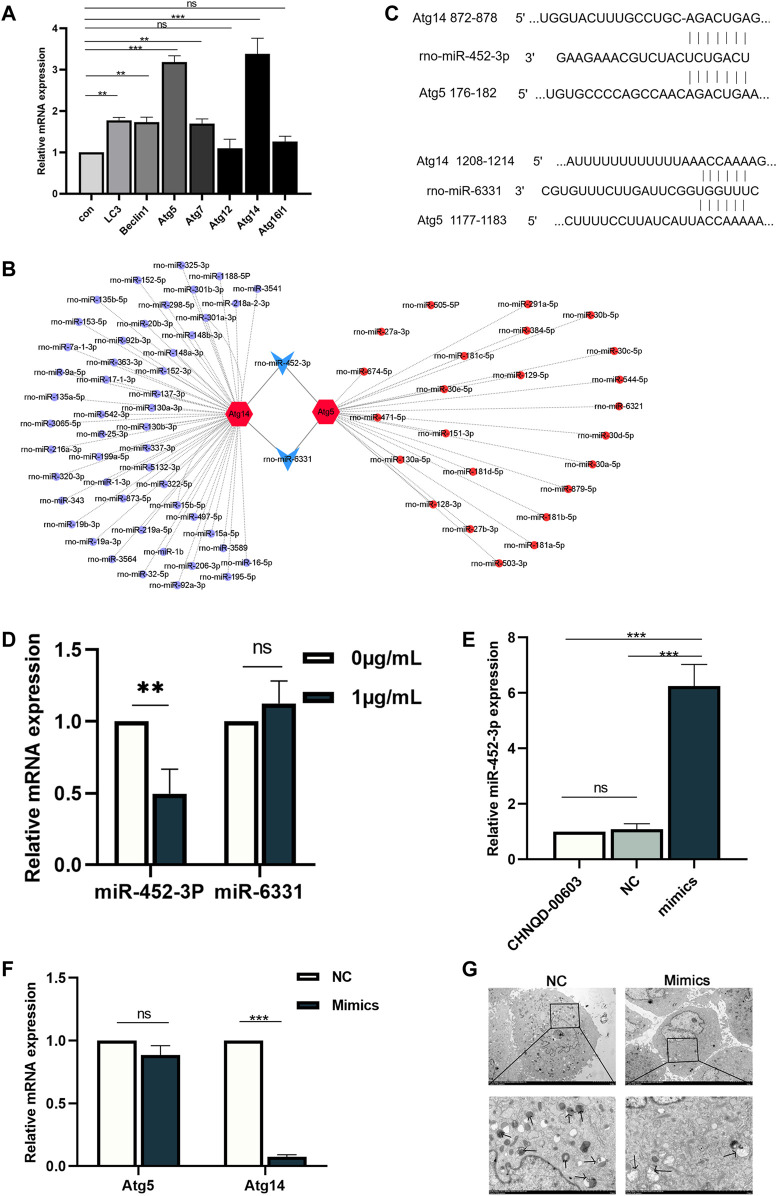
Prediction and validation of potential miRNAs and overexpression of miR-452-3p. **(A)** Autophagy-related genes were estimated by qRT-PCR. **(B)** The targeting network was constructed using Cytoscape. **(C)** The complementary base pairing of miR-452-3p and miR-6331 with Atg5 and Atg14, respectively. **(D)** qRT-PCR was performed to detect the expression of miR-452-3p and miR-6331 in the osteogenesis of BMSCs treated with CHNQD-00603. Upregulation of miR-452-3p attenuates the level of autophagy. **(E)** The efficiency of transfecting miR-452-3p mimics was evaluated by qRT-PCR 48 h after transfecting. **(F)** Autophagy-related genes Atg5 and Atg14 were detected after transfecting miR-452-3p mimics by qRT-PCR on day 7. **(G)** The number of autophagosomes on day 7 was observed by TEM. Quantitative data are presented as the mean ± SD (n = 3) (**p < 0.01, ***p < 0.0001).

We identified the autophagy-related genes (Atgs) by qRT-PCR to further estimate the expression of autophagy. The data showed that the expression of Atg5 and Atg14 was significantly higher than other genes, with three folds that of the untreated group (Figure 6A). Therefore, we presumed that there might be a factor regulating the expression of these two genes. Recently, interactions have been reported between miRNA and autophagy in bone homeostasis (Shen et al., 2016). Therefore, we supposed that CHNQD-00603 induces osteogenic differentiation of BMSCs by regulating miRNAs, which changed the level of autophagy by silencing the target gene. Hence, we selected Atg14 and Atg5 as target genes based on the expression level of the autophagy-related gene. Then, we searched the miRNAs that bind to Atg14 and Atg5 using the miRDB database. The results showed 51 miRNAs with binding sites for Atg14 and 26 miRNAs with binding sites for Atg5 (Figure 6B). Among these miRNAs, we found that miR-452-3p and miR-6331 could bind to Atg14 and Atg5 (Figure 6B). The complementary base pairing was shown in Figure 6C. Therefore, we hypothesized that miR-452-3p and miR-6331 regulate the expression of autophagy in this study. Subsequently, we performed qRT-PCR to validate the expression of these two miRNAs. QRT-PCR assay indicated that miR-452-3p decreased to 0.5-fold in CHNQD-00603-induced osteogenic differentiation of BMSCs compared to the untread group. However, there was no modest change in the level of rno-miR-6331 (Figure 6D). These data suggested that miR-452-3p might participate in the regulation of CHNQD-00603-induced osteogenic differentiation of BMSCs.

### Overexpression of miR-452-3p Decreases the Level of Autophagy

We transfected CHNQD-00603-treated BMSCs with miR-452-3p mimics and NC to explore the effect of miR-452-3p on autophagy. After 48 h, the qRT-PCR data showed that the expression of miR-452-3p had increased six folds in BMSCs transduced by miR-452-3p mimics compared with the NC and controls (Figure 6E). Then we further detected the level of autophagy by qRT-PCR and transmission electron microscopy (TEM). The results revealed that miR-452-3p mimics decreased the expression of Atg14 to 0.1-fold compared with NC, but the downregulation of Atg5 was not different (Figure 6F). Accordingly, the number of autophagosomes decreased significantly (Figure 6G). Thus, our findings indicated that miR-452-3p regulated autophagy levels in BMSCs treated with CHNQD-00603.

### CHNQD-00603 Promotes Osteogenic Differentiation by miR-452-3p Medicating Autophagy

**FIGURE 7:**
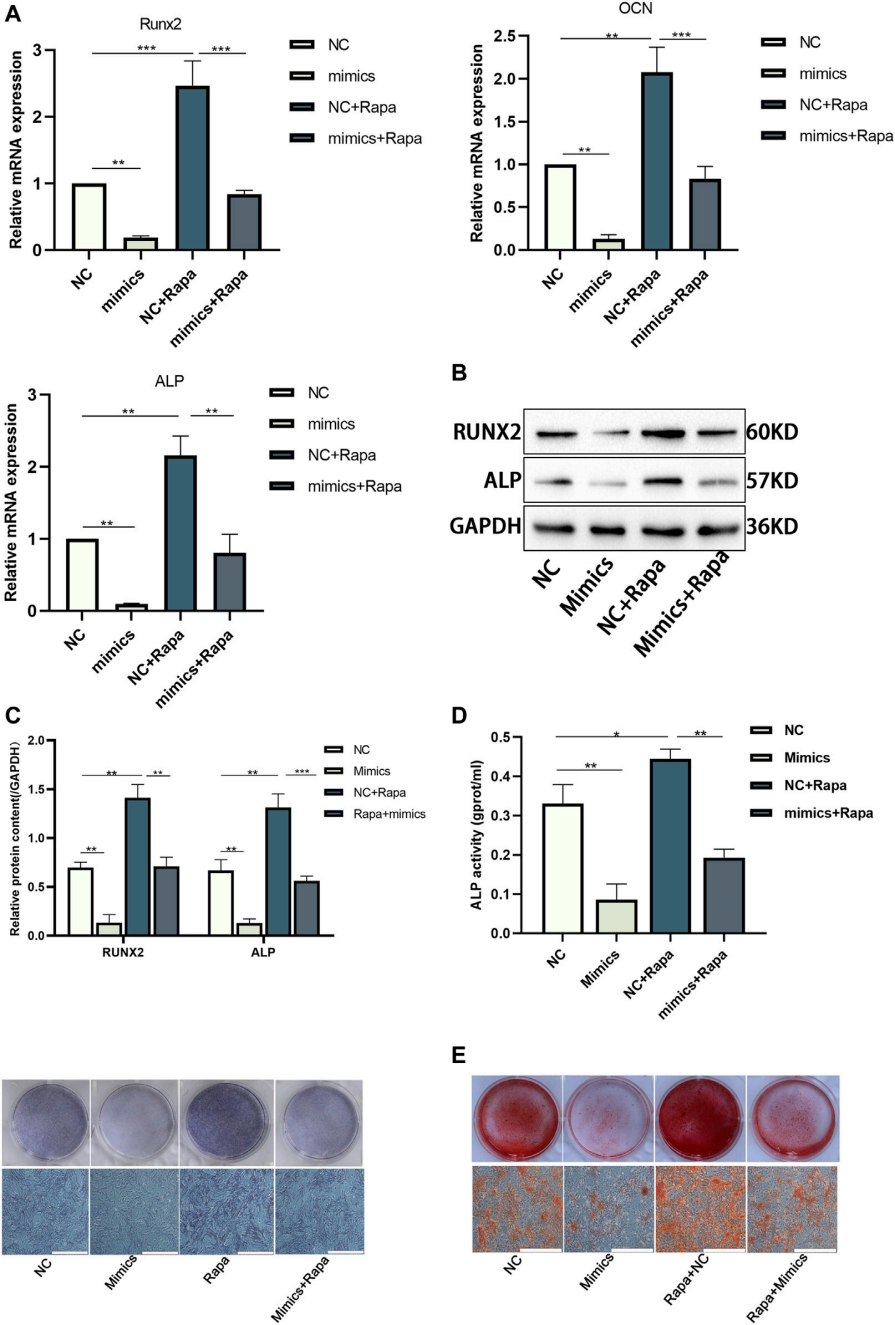
The osteogenic differentiation elevated by Rapa was further disturbed by transfecting miR-452-3p mimics in BMSCs treated with CHNQD-00603. BMSCs treated with CHNQD-00603 were divided into four subgroups: NC, mimics, NC + Rapa, and mimics + Rapa. **(A–C)** Osteogenesis-related genes and proteins were detected by qRT-PCR and Western blot and protein quantification on day 7. **(D)** ALP activity assay and ALP staining were applied to evaluate the early osteogenic differentiation (scale bar = 200 µm). **(E)** ARS was used to estimate calcium salt deposits (scale bar = 200 µm). Quantitative data are presented as the mean ± SD (n = 3) (*p < 0.05, **p < 0.01, ***p < 0.0001).

We used miR-452-3p mimics and Rapamycin in BMSCs cultured with CHNQD-00603 to verify whether osteogenic differentiation induced by CHNQD-00603 was mediated by miR-452-3p regulating autophagy. The results of qRT-PCR and Western blot revealed that transfection of miR-452-3p mimics attenuated the expression of osteogenesis-related mRNA (Runx2, ALP, and OCN) and protein (Runx2 and ALP). In addition, the increased expression of these genes and proteins by Rapa was inhibited by miR-452-3p mimics (Figures 7A–C). ALP activity and ARS consistently showed the same tendency as qRT-PCR and western blotting (Figures 7D, E). Thus, our findings indicated that miR-452-3p targeted autophagy to regulate CHNQD-00603-induced osteogenic differentiation of BMSCs.

## Discussion

Marine natural products are abundant concerning source and variety. A class of derivatives can be obtained by structural optimization with different biological activities, including anti-tumor, anti-inflammatory, and bone metabolism effects. In this study, we obtained several new derivatives by adding different groups to different positions of 4-phenyl-3,4-dihydroquinolin-2(1H)-one core. Preliminary cytological experiments indicated that CHNQD-00603 could potentially promote osteogenic differentiation of BMSCs, as evidenced by the increase in osteogenesis-related gene and proteins Runx2 and early osteogenic differentiation marker ALP activity. By bioinformatics, we found a relationship between CHNQD-00603 and bone metabolism. Therefore, we chose CHNQD-00603 for further investigation. The results of cytotoxicity and osteogenic differentiation experiments showed a lower rate of proliferation of BMSCs on day 7 at a concentration of 1 µg/ml. However, the osteogenic differentiation was strongest, evidenced by the results of qRT-PCR, ARS and ALP staining, and ALP activity assay. Therefore, we believe that the decrease in the cell proliferation rate was associated with early osteogenic differentiation. Furthermore, it is believed that dehydrogenase activity was always relatively low during cell differentiation (Hao et al., 2019). These data demonstrated the role of CHNQD-00603 in osteogenesis for the first time. Thus, the present study provided a groundbreaking choice for bone regeneration.

Autophagy, a fundamental process, sequesters intracellular constituents to deliver them to lysosomes that degrade the intracellular components and recycle them into macromolecule precursors (Chang, 2020). Defective autophagy leads to the senescence of mesenchymal stem cells and bone loss (Li et al., 2018; Yang et al., 2020). Thus, autophagy is an indispensable process in osteogenic differentiation. Autophagy-related signaling molecules, mTOR and PI3K-Akt, were hypothesized to be associated with CHNQD-00603 (Figure 3F). Therefore, we investigated whether autophagy participated in the osteogenic differentiation of CHNQD-00603-treated BMSCs. Our data showed that autophagy was activated in CHNQD-00603-treated BMSCs. In addition, the inhibition of autophagy by 3-MA attenuated osteogenic differentiation. Together with our prediction results, these data indicated that autophagy plays a crucial role in the osteogenic differentiation of BMSCs induced by CHNQD-00603. Our study proposed for the first time that derivatives from 4-phenyl-3,4-dihydroquinolin-2(1H)-one could activate autophagy, promoting the osteogenic differentiation of BMSCs. This provided a new direction to study bone regeneration.

Autophagy-related genes, a group of evolutionarily conserved genes, are involved in the formation and regulation of the autophagy process by communicating with intracellular signaling pathways (Lan et al., 2021). We investigated the expression of a series of autophagy-related genes by qRT-PCR. The results revealed that the expression of Atg5 and Atg14 increased significantly in CHNQD-00603-treated BMSCs. Therefore, we believe that CHNQD-00603 activated autophagy mainly by promoting the expression of Atg5 and Atg14. It has been reported that miRNAs are important regulators of autophagy (Huang et al., 2020; Silwal et al., 2020). Moreover, miRNA is involved in osteogenic differentiation by altering autophagy (Liu et al., 2021). However, the effect of miRNAs on CHNQD-00603-induced autophagy and osteogenic differentiation is still mysterious. Here, by searching the miRDB database and visualizing with a Cytoscape, we found two miRNAs with a targeted contact relationship with both Atg14 and Atg5, including miR-452-3p and miR-6331. Further investigation by qRT-PCR showed that the expression of miR-452-3p decreased in CHNQD-00603-treated BMSCs, but the expression of miR-6331 was not different in CHNQD-00603-treated BMSCs compared with non-treated BMSCs. Therefore, we selected miR-452-3P to further explore its role in CHNQD-00603-induced autophagy and osteogenic differentiation.

We overexpressed miR-452-3p by mimics in BMSCs induced by CHNQD-00603 to determine whether miR-452-3p could regulate autophagy in CHNQD-00603-treated BMSCs. We found that the autophagy activity was suppressed, as evidenced by a decreased number of autophagosomes and autophagy-related genes. In particular, the expression of Atg14 was visibly weakened. We changed autophagy activity by mTOR inhibitor (rapamycin) to identify the relationship between osteogenic differentiation and miR-452-3p-regulated autophagy. The results showed that upregulated osteogenic differentiation by Rapa was inhibited by miR-452-3p mimics. We found and identified a new miRNA, miR-452-3p, which inhibited autophagy. CHNQD-00603 promoted osteogenic differentiation by activating autophagy. However, the expression of miR-452-3p was inversely related to the level of autophagy and CHNQD-00603. Therefore, we believe that CHNQD-00603 plays a vital role in the osteogenic differentiation of BMSCs by miR-452-mediated autophagy.

## Conclusion

In this study, we demonstrated for the first time that CHNQD-00603 could promote the osteogenic differentiation of BMSCs. Furthermore, our research showed for the first time that CHNQD-00603 promoted osteogenic differentiation by miR-452-3p-regulated autophagy. Thus, the present study provided a theoretical basis for the clinical application of marine natural products.

## Data Availability

The raw data supporting the conclusions of this article will be made available by the authors, without undue reservation.
